# Consensus statement on the problem of terminology in psychological interventions using the internet or digital components

**DOI:** 10.1016/j.invent.2020.100331

**Published:** 2020-06-02

**Authors:** Ewelina Smoktunowicz, Azy Barak, Gerhard Andersson, Rosa M. Banos, Thomas Berger, Cristina Botella, Blake F. Dear, Tara Donker, David D. Ebert, Heather Hadjistavropoulos, David C. Hodgins, Viktor Kaldo, David C. Mohr, Tine Nordgreen, Mark B. Powers, Heleen Riper, Lee M. Ritterband, Alexander Rozental, Stephen M. Schueller, Nickolai Titov, Cornelia Weise, Per Carlbring

**Affiliations:** a Department of Psychology, SWPS University of Social Sciences and Humanities, Chodakowska 19, 31 03-815 Warsaw, Poland; b Department of Psychology, Stockholm University, Frescati Hagvag 8, 114 19 Stockholm, Sweden; c Department of Counseling and Human Development, University of Haifa, Israel; d Department of Behavioural Sciences and Learning, Linköping University, Linköping, Sweden; e Department of Clinical Neuroscience, Karolinska Institutet, Stockholm, Sweden; f Universidad de Valencia, Valencia, Spain; g CIBER Fisiopatologia Obesidad y Nutrición (CIBEROBN), Instituto Salud Carlos III, Madrid, Spain; h Department of Clinical Psychology and Psychotherapy, University of Bern, Switzerland; i Uiversitat Jaume I, Valencia, Spain; j Department of Psychology, Macquarie University, Sydney, Australia; k Department of Clinical, Neuro and Developmental Psychology, Vrije Universiteit, Amsterdam, the Netherlands; l Faculty of Behavioural and Movement Sciences, Clinical, Neuro- & Developmental Psychology, Vrije Universiteit, Amsterdam, the Netherlands; m Department of Psychology, University of Regina, Regina, Canada; n University of Calgary, Calgary, Canada; o Department of Psychology, Faculty of Health and Life Sciences, Linnaeus University, Växjö, Sweden; p Centre for Psychiatry Research, Department of Clinical Neuroscience, Karolinska Institutet, & Stockholm Health Care Services, Region Stockholm, Sweden; q Center for Behavioral Intervention Technologies, Department of Preventive Medicine, Northwestern University, Chicago, IL, USA; r Division of Psychiatry, Haukeland University Hospital, Bergen, Norway; s Baylor University Medical Center, Dallas, TX, USA; t Center for Behavioral Health & Technology, Department of Psychiatry and Neurobehavioral Sciences, University of Virginia, Charlottesville, VA, USA; u Institute of Child Health, UCL, London, UK; v Department of Psychological Science, University of California, Irvine, Irvine, CA, USA; w Department of Psychology, Division of Clinical Psychology and Psychotherapy, Philipps–University of Marburg, Marburg, Germany

**Keywords:** Internet-delivered interventions, Digital health, Terminology, Internet, Psychotherapy, Consensus statement

## Abstract

Since the emergence of psychological interventions delivered via the Internet they have differed in numerous ways. The wealth of formats, methods, and technological solutions has led to increased availability and cost-effectiveness of clinical care, however, it has simultaneously generated a multitude of terms. With this paper, we first aim to establish whether a terminology issue exists in the field of Internet-delivered psychological interventions. If so, we aim to determine its implications for research, education, and practice. Furthermore, we intend to discuss solutions to mitigate the problem; in particular, we propose the concept of a common glossary. We invited 23 experts in the field of Internet-delivered interventions to respond to four questions, and employed the Delphi method to facilitate a discussion. We found that experts overwhelmingly agreed that there were terminological challenges, and that it had significant consequences for conducting research, treating patients, educating students, and informing the general public about Internet-delivered interventions. A cautious agreement has been reached that formulating a common glossary would be beneficial for the field to address the terminology issue. We end with recommendations for the possible formats of the glossary and means to disseminate it in a way that maximizes the probability of broad acceptance for a variety of stakeholders.

## Highlights

• The field of Internet-delivered interventions faces inconsistent terminology issue.
• Inconsistent terminology results in miscommunication between stakeholders.
• A common glossary is a potential solution to remedy inconsistent terminology issue.
• A diverse taskforce needs to called upon to propose a common glossary.

“Language is the source of misunderstandings.”

(The Little Prince, Antoine de Saint Exupéry)

## 1 Introduction

The emergence of Internet-delivered interventions in the mid-1990s has led to the continuous introduction of an abundance of approaches, methods, and techniques into the area of psychological and behavioral therapeutic and self-help interventions (Andersson et al., 2019b). As is typical of the richness and multifaceted structure of the Internet, these interventions are heterogeneous (Barak et al., 2009), and they differ from each other in numerous ways, including the technological platforms, use of technical features, level of textuality, degree of inclusion of online and offline factors, extent of human versus automatic involvement, professional qualifications of therapists, and other important aspects. On the one hand, this heterogeneity has generated much research and clinical implementations. On the other, as the field is moving to maturity, consideration needs to be given to rationalising the terminology that is used, to ensure consistency and common understanding. To demonstrate the scope of the heterogeneity in wording, we present the results of our review of terms in PubMed. Table 1 contains a list of terms used in scientific publications to designate Internet-delivered intervention procedures. From rarely used terms, such as chat treatment (1 title in PubMed), computer intervention (10) or online counseling (17), to much more prevalent ones, such as Internet-delivered therapy (175), web-based intervention (250), digital health (546), and teletherapy (785), this list provides an insight into the extent of the issue. Numerous terms imply that many different procedures are possible, each perhaps qualified by its distinctive characteristics. In fact, however, reality shows that there is no direct, or even indirect, connection between a term used and its representation, including the type of computer technology, platform or infrastructure, modality, clinical approach, or the type of intervention applied. Moreover, a review of the literature shows that there is no consistency in use of these terms over time and across publication outlets (Andersson et al., 2019a, Andersson et al., 2019b). Adding to the complexity are the differences in journal preferences and explicit instructions to change terms during the review process.

**Table 1:** Examples of title terms used to delineate internet-delivered psychological interventions, including the number of occurrences in titles in PubMed in November 2019.

Term used	Titles in PubMed	Publication title	Citation
Avatar-assisted therapy	3	Avatar-assisted therapy: a proof-of-concept pilot study of a novel technology-based intervention to treat substance use disorders.	Gordon et al. (2017)
Chat treatment	1	Effectiveness of a web-based solution-focused brief chat treatment for depressed adolescents and young adults: randomized controlled trial.	Kramer et al. (2014)
Computer-assisted therapy	15	Computer-assisted therapy for medication-resistant auditory hallucinations: proof-of-concept study.	Leff et al. (2013)
Computer intervention	10	Development and preliminary pilot evaluation of a brief tablet computer intervention to motivate tobacco quitline use among smokers in substance use treatment.	Brown et al. (2017)
Computerized therapy	6	Attitudes towards the use of computerized Cognitive Behavior Therapy (cCBT) with children and adolescents: a survey among Swedish mental health professionals.	Vigerland et al. (2014)
Computer-mediated psychotherapy	1	Psychotherapy in cyberspace: A 5-dimensional model of online and computer-mediated psychotherapy.	Suler (2000)
Computer therapy	11	My care manager, my computer therapy and me: The relationship triangle in computerized cognitive behavioral therapy.	Cavanagh et al. (2018)
Cybertherapy	17	Cybertherapy meets Facebook, blogger, and second life: an Italian experience.	Graffeo and La Barbera (2009)
Digital health	546	Accelerating digital mental health research from early design and creation to successful implementation and sustainment	Mohr et al. (2017)
Digital service	3	Evaluation of the practitioner online referral and treatment service (PORTS): the first 18 months of a state-wide digital service for adults with anxiety, depression, or substance use problems	Titov et al. (2019)
Distance counseling	1	The effectiveness of physical activity monitoring and distance counseling in an occupational setting - results from a randomized controlled trial (CoAct).	Reijonsaari et al. (2012)
Distance therapy	1	Distance therapy to improve symptoms and quality of life: complementing office-based care with telehealth.	Kroenke (2014)
E-aid	1	Effects of e-aid cognitive behavioral therapy for insomnia (eCBTI) to prevent the transition from episodic insomnia to persistent insomnia: study protocol for a randomized controlled trial.	Yang et al. (2019)
E-counseling	7	Effects of self-guided e-counseling on health behaviors and blood pressure: Results of a randomized trial.	Liu et al. (2019)
E-health program	2	Study protocol of a Dutch smoking cessation e-health program.	Stanczyk et al. (2011)
E-mental health	87	User-centered app adaptation of a low-intensity E-mental health intervention for Syrian refugees	Burchert et al. (2019)
Etherapy	4	Evaluating the role of digital intervention design in treatment outcomes and adherence to eTherapy programs for eating disorders: A systematic review and meta-analysis.	Barakat et al. (2019)
E-therapy	27	e-Therapy in primary care mental health.	Orman and O'Dea (2018)
E-mail therapy	3	Internet administered guided self-help versus individualized e-mail therapy: a randomized trial of two versions of CBT for major depression.	Vernmark et al. (2010)
Guided self-help	187	Do nonsuicidal severely depressed individuals with diabetes profit from internet-based guided self-help? Secondary analyses of a pragmatic randomized trial.	Schlicker et al. (2019)
Internet-administered treatment	4	The effects on depression of Internet-administered behavioral activation and physical exercise with treatment rationale and relapse prevention: study protocol for a randomized controlled trial.	Carlbring et al. (2013)
Internet-based treatment	50	Combining attention training with Internet-based cognitive-behavioral self-help for social anxiety: a randomized controlled trial.	Boettcher et al. (2014)
Internet-delivered therapy	175	Evaluating the efficacy of internet-delivered cognitive behavioral therapy blended with synchronous chat sessions to treat adolescent depression: randomized controlled trial.	Topooco et al. (2019)
Internet intervention	81	The internet intervention patient adherence scale for guided internet-delivered behavioral interventions: development and psychometric evaluation.	Lenhard et al. (2019)
Internet-supported therapy	2	Internet-supported versus face-to-face cognitive behavior therapy for depression.	Andersson et al. (2016)
Internet therapy	11	What makes internet therapy work?	Andersson et al. (2009)
Internet treatment	25	Three-year follow-up of insomnia and hypnotics after controlled internet treatment for insomnia.	Blom et al. (2016)
Interapy	4	Efficacy and effectiveness of online cognitive behavioral treatment: a decade of interapy research.	Ruwaard et al. (2011)
Medicine 2.0	20	Transforming patient experience: health web science meets medicine 2.0.	McHattie et al. (2014)
Minimal-contact intervention(s)	7	The efficacy of minimal contact interventions for acute tinnitus: a randomized controlled study.	Nyenhuis et al. (2013)
Online clinical work	1	Myths and realities of online clinical work.	Fenichel et al. (2002)
Online counseling	17	Exploring young people's perceptions of the effectiveness of text-based online counseling: mixed methods pilot study.	Navarro et al. (2019)
Online intervention	129	Online intervention to reduce pediatric anxiety: an evidence-based review.	Santilhano (2019)
Online program	60	Baby steps - an online program promoting the well-being of new mothers and fathers: a study protocol.	Hamilton et al. (2016)
Online psychotherapy	3	Does the quality of the working alliance predict treatment outcome in online psychotherapy for traumatized patients?	Knaevelsrud and Maercker (2006)
Online therapy	13	Delivering solid treatments on shaky ground: feasibility study of an online therapy for child anxiety in the aftermath of a natural disaster.	Stasiak et al. (2018)
Online treatment	29	Psychodynamic online treatment following supportive expressive therapy (SET): therapeutic rationale, interventions and treatment process.	Beutel et al. (2018)
Self-help through the internet	2	Psychodynamic guided self-help for adult depression through the Internet: A randomized controlled trial.	Johansson et al. (2012)
Telecounseling	2	Telecounseling in rural areas for alcohol problems.	Baca et al. (2007)
Telepsychiatry	349	Telepsychiatry use in U.S. mental health facilities, 2010–2017.	Spivak et al. (2019)
Telepsychology	18	Rationale and design: telepsychology service delivery for depressed elderly veterans.	Egede et al. (2009)
Teletherapy	785	Comparison of functional outcomes and treatment cost between a computer-based cognitive rehabilitation teletherapy program and a face-to-face rehabilitation program.	Schoenberg et al. (2008)
Treatment administered through a smartphone application	2	Behavioral activation vs. mindfulness-based guided self-help treatment administered through a smartphone application: a randomized controlled trial.	Ly et al. (2014)
Virtual reality therapy	50	Attitudes towards and familiarity with virtual reality therapy among practicing cognitive behavior therapists: a cross-sectional survey study in the era of consumer VR platforms.	Lindner et al. (2019)
Web-based intervention	250	Efficacy of a web-based intervention to increase uptake of maternal vaccines: an RCT.	O'Leary et al. (2019)

Note. The table contains arbitrarily selected scientific publications in order to provide examples; there is no intention to comment on particular authors or publication outlets.

It is important to note that for numerous reasons, including the interdisciplinary nature of the field, this extensive vocabulary has not been a result of a consensus reached by representatives of the discipline in the course of a structured debate, but rather has been determined individually or at best locally. These determinations could have been influenced by well-established models or approaches, decisions by funding bodies, preferences of consumers and other stakeholders, cultural differences, as well as for arbitrary or biased reasons. As a consequence, the newness of computers, mobile devices, and the Internet alongside the lack of a long history and established tradition have contributed to local and personal lingual inventions. Terms that name interventions such as “e-mental health,” “Internet therapy,” or “online treatment” have been introduced periodically by individual researchers, but have become adopted more broadly as they resonate with others. The lack of consistency in regard to naming Internet-delivered interventions leads to situations in which terms are used in contexts that depart from their original meanings. For instance, “therapy online” was introduced and named by Colón (1996)—one of the pioneers in this area—to refer to a group chat led by a clinical social worker in which several women took part for two or more hours per day (sessions were named “conferences”), seven days per week, over the course of three months, to work on personal issues. While Colón's term has not been repeated letter-for-letter, the alternate term “online therapy” has been frequently and repeatedly used by many (e.g., Stasiak et al., 2018).

This brings us to the language itself. Although the consideration of all the functions that language serves is beyond the scope of this paper, its two purposes need to be emphasized. First, language is used for individual thinking processes, as it actually reflects ways and contents of thinking (Sfard, 2008). That is, unclear, inconsistent, or even inaccurate wording might *reflect* one's incoherent or perplexed understanding as well as *affect* one's intellectual processes, eventually affecting one's understanding, decision-making, or planning. Second, language is a means of interpersonal communication. Broad and unguided uses of terms—technological and psychological alike—can produce problematic scientific discourse, influence what might be reported and how, and lead to erroneous communication and conflicts. Furthermore, and perhaps even more troublesome, unclear and inconsistent discourse seems to be able to affect the narrative of the still-developing field of Internet-delivered interventions. There is a risk that continuing to use ambiguous and incongruous terms might impede the status and the image of the field.

To fulfill their role in facilitating communication, terminology conventions must be broad and agreed upon. The pervasive and accepted terms make discourse understood, accurate, and effective. Defective and incongruous discourse could not only undermine communication due to unclarity but also slow down the conceptualization and development of any given field. Steps to organize the communication in the field of Internet-delivered interventions had been made before. In Ritterband et al., 2003, Ritterband and colleagues proposed a definition of the term “Internet interventions”. A few years later the International Society for Research on Internet Interventions was formed to facilitate collaboration and communication among researchers and stakeholders (Ritterband et al., 2006). This was followed by a direct attempt to introduce lingual order in the area when Barak et al. (2009) suggested preliminary definitions for some forms of Internet-delivered psychological interventions. This initiative, however, has seemed to have had limited impact, as the incongruence in terminology persists. Possible explanations for this may lie in the process of creating the proposition of typology on the one hand, and in its dissemination on the other. For example, although the number of terms currently in operation can objectively be called excessive, the question remains of whether the relevant community perceives it as such. If not, this could mean that the momentum for change is simply not there yet. However, if many share the view that terminology is an issue, it would be necessary to first establish what its implications are specifically and for whom, and then act accordingly. We stand by the position that the answers to these questions and subsequent recommendations for how to proceed should not be provided by a few individuals, but rather should become a product of a structured exchange among people with vast knowledge of the subject (Borgo et al., 2018). Thus, the purpose of this paper was to provide a platform for such debate.

Specifically, we aimed to investigate the following. First, we sought to determine whether there was a consensus among the experts that the field of Internet-delivered psychological interventions indeed recognized the need to move towards more consistent terminology. Second, should there be an agreement, we wanted to establish what the consequences were for all stakeholders, from creators to consumers to policymakers. Finally, we explored possible solutions to mitigate this issue. In particular, we revisited the idea of formulating a common glossary, but strived to identify the barriers that could be encountered in the process and factors that could determine successful implementation.

## 2 Material and methods

Twenty three experts—clinicians and researchers in the field of mental health—were invited to discuss the issue of terminology in Internet-delivered psychological interventions based on their previously expressed interest in the topic during conferences and other exchanges. One person declined participation, not wanting to take a stand on the terminology issue at this time. The remaining 22 experts were in different stages of their careers—from postdoc to professor—and represented multiple countries: Australia, Austria, Canada, Germany, Israel, Norway, Poland, Spain, Sweden, Switzerland, the Netherlands, and the United States. They are all listed under Authors in this paper. To collect comments, facilitate a discussion, and reach a consensus, the Delphi method was used (Dalkey and Helmer, 1963; Danial-Saad et al., 2013). At its core, the technique is based on an iterative process of distributing questions and subsequent responses to experts in at least three rounds (Danial-Saad et al., 2013). These iterations allow the respondents to revise or elaborate on their own statements, as well as comment on those of others. In the final step, the responses are usually statistically aggregated. However, due to the qualitative character of the data in this project, we followed the approach of Rozental et al. (2014) and extracted common themes instead. Specifically, the following steps were completed. First, an initial survey with four open-ended questions was distributed online among the experts. It contained four questions: 1) In your opinion, is there a problem of unclear and/or inconsistent wording in communication regarding Internet-supported psychotherapeutic interventions? 2) If there is, what issues may arise from this problem (e.g., for professionals, students, and the general public)? 3) Would a proposed glossary for designating Internet-supported psychotherapeutic interventions remedy these issues? 4) Is there anything you would like to add regarding this issue? Do you have another proposed solution? Next, we compiled obtained replies and made them available to all experts, asking them to comment on both their own and others' responses. Based on the answers from these two rounds, a consensus statement was drafted which constitutes the Results section of this paper. Subsequently a manuscript was sent out to each participant. Comments on the draft were incorporated and the revised version was distributed for final comments and approval. Eighteen experts responded to the first and seventeen to the second round of questions, and all 22 revised a draft of the paper. The entire process was anonymous except for the final approval of the manuscript.

## 3 Results

### 3.1 Terminology issue

Due to the lack of shared and accepted definitions, Internet-delivered psychological interventions cover the spectrum from unguided self-help interventions to treatments in which patients receive ongoing feedback from a clinician. Currently, a range of terms are used interchangeably: Internet-interventions, e-therapy, online interventions, Internet-delivered interventions, and digital therapy, to name only a few. Inconsistent use goes both ways: different names are used to describe the same procedures, and similar names refer to different formats.

Differences in terminology are also noticeable on a more granular level than the treatment format: in particular, in describing the type (human vs technology-assisted) and amount of support. Currently, we operate with expressions such as guided, unguided, self-guided, or assisted to convey the extent to which a human is involved in the treatment; however, these terms can and do mean different things to both authors and recipients. They do not clearly reflect the nature of a specialist's involvement, which can be comprised of resolving technical problems, answering non-clinical questions, building rapport, supporting clients in gaining a deeper understanding of material, prompting completion, providing clinically informed feedback, and more (Andersson, 2014). Moreover, it is often not clear how much or how often any assistance is provided. Further contributing to the confusion is that interventions are often referred on the basis of the way they are delivered, which results in the introduction of a multitude of terms, such as app-based, virtual reality, and smartphone interventions (cf., Linardon et al., 2019).

#### 3.1.1 Summary

There was a consensus among the experts that terminology used by the field should, where possible, be more consistent. Presently, the nature of the intervention could not be determined based on the name itself.

### 3.2 Consequences of the terminology issue

Terminology inconsistency leads to miscommunication. Operating with a multitude of terms affects the exchanges among researchers and between practitioners and their patients; it also complicates interactions with students, policy makers, the media, and the general public.

#### 3.2.1 Researchers

In the academia, inconsistent terminology in publications is an obstacle when conducting systematic literature reviews and meta-analyses. Comprehensive reviews are contingent upon using the appropriate keywords, but more recently, those are virtually impossible to narrow down (cf., Andersson et al., 2019a). For instance, a review of guided interventions would require using combinations of words such as assisted, guided, and supported without a guarantee of identifying all relevant studies. Thus, the fact that research findings differ so significantly might partially be caused by this inconsistency in wording. Finding proper and—where possible—distinguishable terms for different forms of intervention would allow researchers to adequately assess the efficacy of Internet-delivered psychological interventions. The lack of consistent terminology also impacts communication among researchers in other ways. For example, it slows down the information flow, as it is necessary to read the intervention's description in detail to fully appreciate the specifics of the program. Furthermore, it makes it harder to build professional consortia and apply for funding together, which, in turn, hinders scientific development.

#### 3.2.2 Students

The problem remains significant down the dissemination line. Students—who are the proximal recipients of information in academia—to an even greater extent than researchers face a problem of searching for relevant literature using keywords. Without distinctive definitions—and without the benefit of knowledge that the definitions are not distinctive—they might wrongly assume that there is only one type of intervention delivered via the Internet, make erroneous comparisons among the formats and procedures, and be unable to differentiate between therapy and self-help materials. Moreover, should they want to design and implement Internet-delivered interventions themselves, they might struggle without having clear guidelines on what constitutes their various aspects, such as the level and type of human support. Finally, through their publications, they also contribute to propagating inconsistent terminology.

#### 3.2.3 Clinicians

In therapeutic context, for health professionals, inconsistent terminology impedes recognition of what each format actually involves if they appear under similar terms. This can lead to making inadequate decisions regarding the level of care. If the possible outcomes for patients in Internet-delivered treatments are exaggerated (e.g., by assuming that they are comparable to face-to-face therapy for all conditions), it might result in insufficient care. Alternatively, the effects of these treatments could be underestimated if clinicians perceive them as self-help materials only, or if they see the technology solely as a mode of communication. However, even therapists who are familiar with the general formats might struggle with more specific issues, such as whether they can interact with the program and incorporate it into their treatments, or whether their patients will continue working with them after initiating program use. For example, therapy via video conferencing is still traditional therapy, only delivered via a different channel (although significant and relevant communication factors make videoconferencing to some degree different from traditional face-to-face therapy; e.g., Drago et al., 2016). Yet, using other media, such as chat programs and apps, changes the way the therapy is delivered. Moreover, there are many differences in the methods of providing synchronous versus asynchronous therapy. Similarly, degree of care is very different when it is a self-guided or blended intervention (Vernmark et al., 2019). Each of these options has advantages and disadvantages, and there is respective evidence for each in terms of their usefulness, clinical-, and cost-effectiveness (Topooco et al., 2017). This multitude of intervention options translates into even more labels, and that might generate resistance among the treatment providers. Using Internet-delivered interventions can be challenging, at least initially, as it requires that professionals be trained in different capacities and methods, but having common and clearly defined terminology would undoubtedly help them navigate this new field.

#### 3.2.4 General public

In the same vein, the general public—including patients—might have the wrong idea about what it means to include technology as part of a therapeutic or clinical process. One problem was already mentioned: assuming incorrectly that communication tools, such as Skype, constitute Internet-delivered therapy. Yet, even the word “Internet” can limit the understanding of available options, as it seems to imply the need to sit in front of a computer and does not account for other forms of delivery, such as mobile apps or virtual reality. The multitude of terms makes it difficult to immediately recognize if a given option involves meeting a therapist face-to-face or “just” via the Internet-supported device. This, in turn, might cause reluctance to initiate such interventions or, at the very least, prevent people from making an informed decision regarding their own healthcare. Furthermore, when the terms are not clearly defined, patients might come to have expectations regarding the procedure that are subsequently not met. Finally, the lack of consistently used terminology across the field prevents users from easily distinguishing empirically validated interventions—both therapy with digital components and self-help interventions—from non-evidence-based ones.

#### 3.2.5 Media

An important intermediary between the world of research and therapeutic practice and the general public is the media. Journalists face additional problems when the terminology is unclear: not only do they need to comprehend the terms as they are constructed by academics, but they also must translate them into a language understood by a broad audience. Hence, if there is no consensus on the terminology among researchers, it is hardly possible for the media to report and interpret research findings correctly and communicate an accurate message to the public.

#### 3.2.6 Decision makers

A major group of stakeholders in the field of Internet-delivered interventions constitutes policy makers, funding agencies, and health organizations. As with representatives of other groups, the main problem here is a lack of awareness of what Internet interventions actually involve and in what way their formats and procedures differ. However, in this case, confusion impacts the full circle of a given intervention's lifecycle, from securing funds to development to the dissemination of its results. If decision makers cannot easily differentiate between the more-effective and less-effective treatments, or in what kind of context an intervention is suitable to deliver, they might find it difficult to make informed decisions regarding funding. At the other end of the process, limited or misguided dissemination impacts the uptake among patients.

#### 3.2.7 Summary

There was a consensus among experts that inconsistent terminology in the field of Internet-delivered psychological interventions resulted in disrupted communication among interested parties. This affected funding, research, education, and clinical practice.

### 3.3 Glossary: a potential solution?

The consensus among researchers—as clearly found in our Delphi Study— that there was a problem of terminology, and that it carried significant consequences for numerous stakeholders, raised the question of whether introducing a common glossary could be a useful solution. The short answer is “maybe”. This hesitation seems to be partly generated by foreseen obstacles to first agreeing on common terms, and then ensuring that they are actually used. There are questions regarding the glossary's size, the scope of the terms, and of course the fundamental one: who should stand behind these decisions and subsequently enforce them. The experience, knowledge, and influence of decision makers would undoubtedly be factors in subsequent implementation processes. Therefore, there seems to be an agreement that the glossary should not be a product of a discussion among researchers only. Ideally, it would be a result of consensus among a special “task force” comprised of representatives of all relevant parties, that could include scientists, journal editors, users/patients, policy makers, health care providers, and technical experts such as programmers and user-experience specialists.

Regardless of the specific terms that would eventually comprise the glossary, a shared vision of its structure is that it should allow for distinguishing between interventions on both the general and specific levels, and highlight the similarities and differences between them. For instance, the broader umbrella terms that describe wide-ranging approaches to using digital technologies in therapy would be followed by the lower-level specifiers that refer to the treatment components (e.g., degree of human guidance). Alternatively, the structure could take the form of a continuum that takes into account “how much” technology and therapist support there is in a given treatment. Yet another option is a checklist format (e.g., similar to CONSORT; Grant et al., 2018) containing questions developed to describe the intervention (e.g., Is this a self-help intervention?) and that prompt short, mostly yes/no responses.

For the glossary to succeed, at least partly, in solving the terminology issue, it needs to be followed by a proper and wide-scale dissemination. The first challenge in the implementation process is that—in a manner typical of all taxonomies—older definitions tend to prevail for quite some time. Thus, the process of introducing the new terminology (or perhaps eliminating the old one) might be slow. This issue is potentially exacerbated by the reality of rapid technological development, which makes some terms obsolete relatively soon after their introduction. Thus, systematic updates of the glossary would be necessary. Its survival depends on promotion across disciplines and national contexts. An example of how the latter can pose a challenge is a current tendency to call similar procedures Internet interventions in continental Europe, computerized cognitive behavioral therapy (CBT) in the United Kingdom, digital health in the United States, and digital/virtual health services in Australia. The dissemination even faces a time-related dilemma, as it remains an open question what to do with the past publications. As inconsistent terminology could have hindered or misdirected developments in the past, in both practice and research, is there a need (and if so, a way) to apply a common terminology to what has already been published?

A few factors could facilitate the successful dissemination. First, it is important that frontrunners across the fields endorse the common terminology. It could start with them forming the above-mentioned task force. Their consistent use of the common glossary in official communication—within and outside of academia—would inspire others to follow. Second, using the glossary should be strongly recommended by publication outlets and incorporated into their guidelines for authors. This could lead to a wider adoption of the terms: if they are used in research reports, they would later become part of systematic reviews and meta-analyses, which would subsequently validate their usefulness by mapping them to empirical data, and probably also spur a few updates. Third, a related solution is to create a glossary website that could be linked to by the journals, professional organizations, licensing authorities, government agencies, ethical codes, and academic institutions. The advantage of this solution is that only a single information source would need to be updated when necessary. Fourth, the consensus on the terminology, along with its rationale, could be presented at key professional conferences. This would have an additional benefit of being useful for addressing comments and answering questions from the community. Furthermore, organizations behind these conferences could endorse the proposed glossary. Finally, wide usage could be modelled by researchers and practitioners consistently applying these terms in all contexts (e.g., scientific and non-scientific publications, education, appearances in the media, and communication with policymakers).

#### 3.3.1 Summary

The idea of creating a common glossary received moderate support among the experts. While there was a consensus that an attempt to develop a glossary should be made, its success was perceived as contingent upon three factors. First, members of the “task force” called upon to propose a typology need to represent diverse environments in terms of culture and occupation. Second, the structure of the typology would need to be easy to update and allow for both general concepts and their more specific components: Network of concepts is preferable over static dictionary. Finally, the implementation process would have to encourage wide-scale use of the common glossary.

## 4 Discussion

Since its origin, the field of Internet-delivered interventions has generated plenty of terms to describe the treatment formats and their components. Over time, this has progressed to a situation that experts in the discipline agree needs attention. This state of affairs is perhaps understandable, given the relatively recent emergence of the technologies that enabled the discipline and the variability across their applications. It is possible that over time some specific terms will eventually dominate the field and the number of operating terms will shrink. On the other hand, it is likely that the opposite will happen or that the terms will simply change (Eysenbach, 2019). It is our position that the consequences of inconsistent terminology, as they were presented in this paper, more than justify calling for action now.

We had three aims with this project. The first was to establish whether the experts in the field of Internet-delivered interventions agree that we face a pressing issue with inconsistent terminology. The answer to that was a resounding “yes.” The second aim was to investigate the implications of this issue. While we could predict that they would be predominantly related to communication, we wanted to be able to specify what consequences exist for which party. In the process of having a structured discussion, we were able to extract the difficulties caused by the inconsistent terminology. Some of these difficulties were typical for research (e.g., conducting a literature review using adequate keywords), and some were relevant for more than one group of stakeholders (e.g., differentiating between using technology as a part of intervention versus only as a mode of delivery). It is worth noting that the areas for which we identified implications of inconsistent terminology were determined through responses to the open-ended questions posed to the experts, and were not pre-determined. Specific areas (i.e., research, education, practice, media and policy making bodies) emerged only during the subsequent structured discussion. Moreover, the consequences listed in this paper are not exhaustive; In order to fully review all potential implications for each of these areas a thorough and directed examination would have to be conducted. Similarly, probably not all issues stemming from inconsistent terminology were raised. For example, we found that experts emphasized the degree of human support in Internet-delivered interventions, which might indicate that this is a particularly pressing problem, however other aspects in this field—such as descriptions of interventions in terms of how gamified or persuasive their designs are—also seem to suffer from ambiguity in terminology. Finally, the third goal was to investigate whether introducing a common glossary would constitute a solution to the problem. The answer to this question turned out to be complex and dependent upon whether a set of conditions can be met, including finding an adequate format for the glossary and taking steps to encourage its use on a large scale.

Based on these findings, we propose that the next step should be to form a team of experts—a “task force”—comprised of representatives of areas across the field and from different cultural contexts. The need for such a group could be communicated via this very paper, editorials in leading journals in the field, at conferences such as the International Society for Research on Internet Interventions (ISRII), and through informal channels. Diversity in terms of expertise, nationality, and career stage seems to be the key to gaining a vote of confidence. Results of the proceedings of this group, including the glossary's proposal and implementation plan, could be disseminated in the form of a follow-up consensus statement.

It is inevitable that whatever set of terms is agreed upon, it will not be accepted by all relevant actors. This is understandable, as it is hardly possible to create a glossary that would account for the heterogeneity throughout the field. For example, terminology will most probably prevail to differ to some extent by region and across domains. In fact, one limitation of this study is that responders were predominantly researchers in clinical psychology and came from global North. In the process of proposing the glossary, greater diversity will be needed. Yet, the benefit of better communication between representatives of various groups likely outweighs the cost of an imperfect initial glossary. Creating the first typology opens up a possibility of perfecting it over time, or, alternatively, agreeing that a finite vocabulary is not a goal, and instead, that it is important to have an efficient updating procedure in place. At the same time, a common glossary has not been universally valued as a solution to all the issues that we listed in this paper, and as a professional community, we should remain open to other ideas.

Because the challenges related to inconsistent language reflect the emerging maturity of the field, we appreciate that there is already enough research to even warrant a discussion of terminology. Despite the arguments laid out above to demonstrate how heterogeneous the vocabulary is and what impact it has on the development of the discipline, the discipline seems to move forward nevertheless, producing research, organizing societies, and attracting new adepts among both academics and practitioners. On the one hand, this suggests that there is no telling how far we can go when we start speaking the same language. On the other, perhaps, not having a common terminology for Internet-delivered psychological interventions constitutes a problem not only because of all the negative consequences listed in this article, but because of the positive aspects of having one. Having common terms would facilitate the dissemination of Internet-delivered interventions. An analogy can be drawn to the terminology used for CBT. The term CBT is widely accepted by clinicians and researchers despite the fact that CBT actually is an umbrella term and the specific therapeutic techniques used can be different. In fact, several similar terms could be used instead without hindering the communication between interested parties. However, having a label recognized by everyone helps to include all research and knowledge within that umbrella, accumulate evidence, better disseminate this type of intervention, better identify this field of work, and give greater visibility to a field. It creates a “brand,” which makes it easier to recognize a product, and is even perceived of as a seal of approval. Having a common term for psychological interventions conducted with the aid of technology could help to achieve the same goals.
